# Ethnic and geographical differences in HLA associations with the outcome of hepatitis C virus infection

**DOI:** 10.1186/1743-422X-6-46

**Published:** 2009-05-01

**Authors:** Jane H Wang, Xin Zheng, Xiaogang Ke, M Tevfik Dorak, Jingjing Shen, Basmattee Boodram, Maurice O'Gorman, Kenneth Beaman, Scott J Cotler, Ronald Hershow, Lijun Rong

**Affiliations:** 1 Division of Hepatology, Department of Medicine, University of Illinois at Chicago, IL, USA; 2 Division of Gastroenterology, Hepatology and Nutrition, Department of Pediatrics, the Feinberg School of Medicine, Northwestern University, Chicago, IL, USA; 3 Clinical Immunology Laboratory, Rosalind Franklin University of Medicine and Science, Chicago, IL, USA; 4 Genomic Immunoepidemiology Laboratory, HUMIGEN LLC, The Institute for Genetic Immunology, Hamilton, NJ, USA; 5 Division of Epidemiology and Biostatistics, School of Public Health, University of Illinois at Chicago, Chicago, IL, USA; 6 Division of Clinic Immunology, Department of Pediatrics, the Feinberg School of Medicine, Northwestern University, Chicago, IL, USA; 7 Department of Microbiology and Immunology, University of Illinois at Chicago, Chicago, IL 60612, USA; 8 B&H Biotechnologies, Willowbrook, IL 60527

## Abstract

**Backgrounds:**

The association of human leukocyte antigen (HLA) genes with the outcome of hepatitis C virus (HCV) infection may be modified by ethnic and geographical differences.

**Results:**

HLA-A, -C, -DRB1 and -DQB1 genotyping were performed in a Midwestern American cohort of 105 HCV infected subjects among which 49 cleared HCV infection and 56 had persistent viral infection. A new protective association of HLA-Cw*05 to HCV infection of all ethnic populations was identified (OR = 0.12, 95% CI = 0.01–0.97, P = 0.03). It was surprising that HLA-A*02 (*P *for interaction = 0.02) and HLA-DRB1*12 (*P *for interaction = 0.05) showed statistical interaction with race indicating opposite associations in Caucasians (OR = 2.74 for A*02 and 2.15 for DRB1*12) and non-Caucasians (OR = 0.41 for A*02 and 0.15 for DRB1*12). In addition, HLA-DRB1*01 (OR = 0.26), DQB1*05 (OR = 0.23) and the haplotype DRB1*01-DQB1*05 (OR = 0.19) showed strong associations with viral clearance in Caucasians. The protective associations of A*03 (OR = 0.20) and DQB1*03 (OR = 0.20) were exclusive to non-Caucasians. In contrast, DQB1*02 (OR = 2.56, 95% CI = 1.15–7.71, P = 0.02) and the haplotype DRB1*07-DQB1*02 (OR = 5.25, 95% CI = 1.04–26.6, P = 0.03) were risk markers in Caucasians.

**Conclusion:**

The associations of HLA-A*02 and HLA-DRB1*12 with HCV infection are opposite with different races. HLA-A*03, Cw*05, DRB1*01, DQB1*03 and DQB1*05 are associated with viral clearance while HLA-DRB1*07 and DQB1*02 are risk markers for viral persistence of HCV infection in Midwestern Americans. These results reveal ethnically and geographically different distribution of HLA-genes which are associated with the outcome of HCV infection.

## Backgrounds

Hepatitis C virus (HCV) has infected at least four million Americans and more than 200 million individuals globally. Over 70% of the patients develop chronic infection leading to end stage liver diseases and, in many cases, death [1,2]. Cellular immunity is believed to play a central role in the host control of HCV infection [3-12]. To elicit an adaptive cellular immune response, HCV antigens are processed into peptides that bind human leukocyte antigen (HLA) molecules. These are then presented on the cell surface of either antigen presenting cells or on infected cells such as hepatocytes. CD8 or CD4 T cells can recognize the complex of HLA- class I peptides or class II peptides and act as either effector T cells, helper T cells, or regulatory T cells through direct killing, cell-contact or by secreting cytokines, respectively [3,5-9,13].

It has been reported that HLA-alleles are associated with the outcome of HCV infection. The majority of such studies have been focused on the associations between HLA-class II alleles and HCV infection. In addition, the reported associations showed ethnic and geographical differences [14-20]. One study of a Northeastern American population in Baltimore showed that HLA-DQB1*03 is protective in African Americans but not in Caucasians [18]. The results of another study of Northeastern Americans in Philadelphia conversely suggested that DQB1*03 is protective in Caucasians but not in African Americans [14]. Therefore, further study on ethnic and geographical differences in HLA associations with the outcome of HCV infection is needed.

We have identified HCV-infected subjects through a cohort that enrolled young injection drug users with a defined date of HCV acquisition in Chicago area, Illinois. In the present study, we show that HLA association with the outcome of early HCV infection is significantly different among different races.

## Results

### Subjects with HCV infection

One hundred and five subjects who were exposed to HCV in the Chicago area were divided into two groups: group 1 included 56 patients with persistent HCV infection and group 2 included 49 subjects with spontaneous viral clearance (Table 1). Chronic HCV infection was determined by persistently positive detection of HCV-RNA and antibodies in the blood samples collected at each patient visit during the cohort. Spontaneous viral clearance was determined by detectable HCV antibodies, undetectable HCV-RNA and consistently normal values of serum alanine aminotransferase. In statistical comparisons, the persistent HCV-infected group was treated as cases and the clearance group as controls.

**Table 1:** Characteristics of study subjects

HCV	Race (%)		Gender (%)		Age	Inf time (yrs)	Total
					
Infection	Cauc	non-Cauc	Male	Female	Mean (range)	Mean (range)	(n)
Persistence	33 (58.9)	23 (41.1)	33 (58.9)	23 (41.1)	25.6 (19~32)	2.5 (0.1~6.0)	56
Clearance	23 (46.9)	26 (53.1)	29 (59.2)	20 (40.8)	26.0 (19~33)	2.6 (0.1~6.6)	49

Note: Cauc: Caucasian; Inf: infection; yrs: years; n: number.

Caucasians comprised 46.9% of the clearance group and 58.9% of the persistent group, respectively. A trend of susceptibility for Caucasians was observed (OR = 1.62, 95% CI = 0.75–3.52, P = 0.22). Among the non-Caucasian population, 60~65% was Hispanic Latino American, 23~30% was African American, 7~9% was mixed races and 3% was others (Table 1). There were no differences in age, gender or estimated years of infection between the two groups which could have contributed to the observed differences (Table 1).

### Associations of HLA-class I alleles with HCV infection

The frequencies of HLA-A and -C alleles are shown in Table 2. A trend of risk association of HLA-A*01 (OR = 2.41) was observed among the Caucasian population, but this was not statistically significant. HLA-A*02 showed opposing trends in different ethnic groups: risk for Caucasians (OR = 2.74) and protective for non-Caucasians (OR = 0.41). Although these associations were not statistically significant, an interaction with race (the difference between the ORs for each race) was evident (*P *for interaction = 0.02). Protective associations of HLA-A*03 to HCV infection with high dose viral inoculation has been reported in European Caucasians [18]. Our data indicate that A*03 is significantly protective for non-Caucasians (OR = 0.20) but not for Caucasians (Table 2). HLA-A*11 in Northeastern American Caucasians in Baltimore has been reported to be protective for HCV clearance [18]. In our study, A*11 (OR = 0.55) demonstrated a weak trend of protective association (Table 2).

**Table 2:** HLA-class I allele frequencies of HCV infected subjects

Allele	Chronic(%) 2n = 112	Clearance (%) 2n = 98	Odds Ratio	95% CI	p-value
A*01	15.2	9.18	2.05	0.79–5.32	0.14
A*02	26.8	26.0	1.04	0.48–2.24	0.92
Caucasian	30.3	17.4	**2.07**	0.82–5.21	0.12
non-Cauc	21.7	32.7	**0.57**	0.23–1.42	0.23
A*03*	9.82	16.7	0.54	0.24–1.20	0.14
A*11	3.57	6.25	0.55	0.15–2.08	0.38
A*23	1.79	4.17	0.42	0.07–2.38	0.33
A*24	10.7	15.0	0.94	0.37–2.38	0.90
A*25	1.79	1.25	1.78	0.16–20.2	0.64
A*29	5.34	1.25	0.18	0.02–1.50	0.12
A*30	7.14	3.12	2.56	0.64–10.2	0.19
A*31	3.57	5.21	0.68	0.17–2.68	0.58
A*32	1.79	4.17	0.42	0.07–2.38	0.33
A*33	2.68	1.04	2.71	0.27–27.0	0.39
A*68	2.68	4.17	0.64	0.14–2.08	2.99
A*74	2.68	1.04	0.37	0.04–3.66	0.62
					
Cw*01	1.79	4.08	0.57	0.09–3.55	0.55
Cw*02	9.84	7.16	1.47	0.52–4.13	0.47
Cw*03	10.7	9.18	1.21	0.46–3.18	0.70
Cw*04	16.9	13.2	1.31	0.56–3.06	0.53
Cw*05	0.89	7.16	**0.12**	**0.01–0.97**	**0.03**
Cw*06	8.94	9.18	1.11	0.40–3.09	0.84
Cw*07	28.6	28.6	0.88	0.41–1.91	0.75
Cw*08	4.46	3.06	1.18	0.25–5.55	0.84
Cw*12	3.58	6.12	0.55	0.15–2.08	0.38
Cw*15	3.58	2.05	1.81	0.32–10.3	0.51
Cw*16	7.15	8.16	0.85	0.29–2.48	0.77

*: A*03 is protective for non-Caucasians, OR = 0.20, 95% CI = 0.04–1.01, P = 0.04

Note: Only those alleles except Cw*05 with more than 1.0% frequency in both comparison groups are shown.

Non-Cauc: non-Caucasians.

A weak protective association of Cw*05 has been observed in European Caucasians[15]. Surprisingly, HLA-Cw*05 frequency in chronic infection and viral clearance is 0.89% versus 7.14%, respectively, suggesting a strong protective association of Cw*05 and HCV resolution (OR = 0.13). A strong protective association of Cw*01 to HCV clearance has been reported in European and Northeastern American Caucasians in Baltimore [15,17]. Our data show that the allele frequencies of Cw*01 in chronic infection and viral clearance is 1.79% versus 4.08%, respectively, but this difference is not statistically significant (Table 2).

The risk association of Cw*04 with viral persistence has been reported in both Caucasians and non-Caucasians of Northeastern Americans in Baltimore [15,17]. Neither protective nor risk associations of the two alleles with HCV infection were observed in either Caucasians or non-Caucasians in the present study.

### The associations of HLA-class II alleles with HCV infection

The protective associations of DRB1*0101 and DQB1*0501 have been documented in Northeastern American Caucasians in Baltimore whereas in African Americans, viral clearance is mainly associated with DQB1*0301 [18]. Our data confirmed that both DRB1*01 and DQB1*05 had a strong association with viral clearance in Caucasians (Table 3). Consistently, HLA-DQB1*03 protective association was exclusive to non-Caucasians (Table 3). Among the non-Caucasian subjects involved in our study, 60~65% were Hispanic Latino Americans and 23~30% were African Americans. The frequencies of DQB1*03 in Hispanic Latino Americans and African Americans are 50% and 33.3%, respectively. Thus our data, as well as others' findings, indicate that the DQB1*03 protective association is equally strong in African Americans [18] and Hispanic Latino Americans.

**Table 3:** HLA-class II allele frequencies of HCV infected subjects

Allele	Chronic (%) (2n = 112)	Clearance (%) (2n = 98)	Odds Ratio	95% CI	p-value
DRB1*01	6.25	14.6	**0.37**	**0.12 – 1.08**	**0.07**
Caucasian	7.58	19.6	**0.27**	**0.13 – 1.00**	**0.05**
DRB1*03	14.3	10.4	1.56	0.63 – 3.86	0.34
DRB1*04	13.4	18.7	0.83	0.35 – 1.98	0.68
DRB1*07	17.0	8.35	**2.42**	**0.95 – 6.23**	**0.07**
DRB1*09	1.60	2.08	0.87	0.12 – 6.24	0.89
DRB1*11	10.7	12.5	0.84	0.34 – 2.09	0.71
DRB1*12	4.46	6.25	0.70	0.20 – 2.46	0.58
DRB1*13	16.1	9.38	1.62	0.64 – 4.12	0.31
DRB1*14	3.70	8.35	0.46	0.12 – 1.68	0.24
DRB1*15	9.82	6.25	1.75	0.60 – 5.15	0.31
DRB1*16	2.67	2.08	1.33	0.21 – 8.31	0.76
					
DQB1*02	25.9	16.3	1.79	0.91 – 3.54	0.09
Caucasian	34.8	15.2	**2.98**	**1.15 – 7.71**	**0.02**
DQB1*03	28.6	40.8	**0.39**	**0.18 – 0.92**	**0.02**
non-Cauc	23.9	44.2	**0.20**	**0.06 – 0.67**	**0.01**
DQB1*04	5.40	2.10	4.24	0.76 – 23.6	0.10
DQB1*05	10.7	21.4	**0.42**	**0.18 – 1.01**	**0.05**
Caucasian	9.10	23.9	**0.23**	**0.07 – 0.82**	**0.02**
DRB1*06	29.4	19.4	1.72	0.79 – 3.76	0.17

Note: only alleles with more than 1.0% frequency in both comparison groups are shown. non-Cauc: non-Caucasian.

We found strong linkage disequilibrium (LD) between HLA-DRB1*01 and DQB1*05 (Δ = 0.0419, P = 0.0009) as expected. Because of strong LD, co-occurrence of these two alleles in the same individual was considered as a haplotype. Logistic regression analysis demonstrated that there is a strong protective association with the haplotype DRB1*01-DQB1*05 (OR = 0.20). This is statistically significant in Caucasians only (OR = 0.19). The DRB1*01-DQB1*05 haplotype association remains statistically significant in combination with all other markers as shown by multivariable logistic regression analysis (OR = 0.11, 95% CI = 0.27 to 0.46, P = 0.002). Thus, it appears to be the primary association. This result is consistent with another study performed on Northeastern Americans in Baltimore [18].

The DQB1*02 yielded high OR (1.8) for risk association in combined ethnic populations. Consistent with others reports [15,18], the association was stronger in Caucasians (OR = 3.0, Table 3) and not significant in non-Caucasians. The haplotypic association of DRB1*03-DQB1*02 has been reported in both European and American Caucasians [15,18] but it was not observed in our study. Interestingly, the co-occurrence of DRB1*07-DQB1*02 (OR = 5.25) was very significant in Caucasians. Strong linkage disequilibrium between DRB1*07 and DQB1*02 (Δ = 0.0351, P = 0.005) suggests that DRB1*07-DQB1*02 occurred as a haplotype.

HLA-DRB1*07-positive individuals appeared to be susceptible to chronic HCV infection (OR = 2.42). When stratified by race, the risk association with DRB1*07 was stronger in Caucasians (OR = 3.81, Table 3). This association has also been reported in European Caucasians [15]. However, the results of multivariable logistic regression analysis indicated that DRB1*07 association did not remain significant in the presence of other associated markers (Cw*05, DRB1*01-DQB1*05, DQB1*03). This suggests that DRB1*07 association was mediated through the reciprocal effect of the others and it is not a primary association.

DRB1*13 showed a trend of risk association in combined ethnic population (OR = 1.62), which was stronger in non-Caucasians (OR = 2.61) (Table 3). DQB1*04 yielded a very high OR (4.23) in non-Caucasians which was not statistically significant (Table 3).

DRB1*12 was another allele whose positivity showed opposing associations in Caucasians (OR = 0.15) and non-Caucasians (OR = 2.53, both were non-significant). As with HLA-A*02, however, the interaction test yielded statistical significance for the difference of the odds ratios (*P *= 0.05). Thus, this is another example of effect modification of an HLA association by race.

### Multivariable model

A multivariable model consisting of all markers that yielded OR < 0.5 or OR > 2.0 was constructed. In this model, only the co-occurrence of DRB1*01-DQB1*05, DQB1*03 and race remained independent associations. The adjusted ORs were 0.11 for DRB1*01-DQB1*05; 0.25 for DQB1*03 and 2.40 for Caucasians.

### Protective and risk associations of HLA- alleles with HCV infection

Taken together, our results suggest the HLA-gene controlled associations to HCV infection differed among different ethnic populations and geographical locations. In Table 4, we present the protective and susceptibility associations of HLA-alleles with HCV infection of Europeans and Americans found by others and in the present study. HLA-A*11, B*57 are protective and Cw*04 confers risk to HCV infection in both American Caucasians and African Americans. HLA-A*03 is protective for European but not American Caucasians. In addition, the protective association of A*03 to viral clearance is significant in non-Caucasians (Tables 2 and 4). Furthermore, the strong association of Cw*05 in combined ethnic populations suggests that this allele protects both American Caucasians and non-Caucasians.

**Table 4:** Protective and susceptibility HLA-alleles in different races

	Caucasians		non-Caucasian Americans*		
			
Allele	European	American	AA	HL	Reference
**Protective**					
A*02			Chicago	Chicago	Present
A*03	Irish**		Chicago	Chicago	15, Present
A*11		Baltimore	Baltimore		17
B*27	Irish				15
B*57		Baltimore	Baltimore		17
Cw*01	Irish	Baltimore			15, 17
Cw*05	Chicago	Chicago	Chicago		Present
DRB1*01	Irish	Baltimore, Chicago			15, 17, present
DRB1*11	British, Italian	Philadelphia			14, 16, 19
DRB1*12			Chicago	Chicago	Present
DQB1*03	British	Philadelphia	Baltimore		14, 18, 19
		Chicago	Chicago		Present
DQB1*05	Irish	Baltimore, Chicago			15, 18, 19
**Susceptibility**					
A*02		Chicago			Present
A*23				Baltimore	17
Cw*04		Baltimore	Baltimore		17
DRB1*03	Irish, German	Baltimore			15, 18, 20
DRB1*07	British	Chicago			19, present
DRB1*12		Chicago			Present
DQB1*02	Irish	Baltimore, Chicago			15, 17, present

*: All Americans are injection drug users; AA: African American; HL: Hispanic Latino American. **: Irish females. Sample size (n): Irish: 227; British: 250; Baltimore:548/675; Chicago: 105; Philadelphia: 93; German: 75; and Italian: 73.

Many of the associations, however, are related to race. Protective associations of Cw*01, DRB1*01, DRB1*11 and DQB1*05 and risk associations of A*01, A*02, DQB1*02 and DRB1*07 are significant only in Caucasians (Table 4). The association of HLA-DQB1*03 to HCV clearance are affected by geographical location. Two reports, including ours, indicate that the association is significant only in non-Caucasian Americans while two other studies show that it is significant in American and European Caucasians but not in African Americans (Table 4). Overall, it appears that risk associations of especially class II alleles are less common in non-Caucasians (Table 4).

## Discussion

The current study investigated the associations of HLA-class I and -class II alleles to the outcome of early HCV infection in a Midwestern American population. Similar to other two studies of Northwestern Americans in Baltimore and Philadelphia, subjects of this study were participants of a cohort that enrolled injection drug users in the Chicago area with defined dates of HCV acquisition, viral loads, serum levels of alanine aminotransferase and ethnicity. Our data confirmed some of the results from the study on Northeastern Americans in Baltimore [17,18].

A new protective association of HLA-Cw*05 and a risk association of haplotype DRB1*07-DQB1*02 to HCV infection were identified in the present study. In addition, the DQB1*03 was found to be significantly protective in non-Caucasians. It is interesting that our study revealed the modification of an HLA association by race. HLA-A*02 showed opposing trends in different ethnic groups: risk for Caucasians (OR = 2.74) and protective for non-Caucasians (OR = 0.41) DRB1*12 was another allele whose positivity showed opposing associations in Caucasians (OR = 0.15) and non-Caucasians (OR = 2.53) and the interaction test yielded statistical significance for the difference of the odds ratios for both A*02 and DRB1*12 (*P *< 0.05).

Our results and other studies demonstrate associations of genes encoding HLA-class I antigens with the outcome of HCV infection (Table 4). Some associations are race and geography dependent. For example, a strong protective association of Cw*01 to HCV clearance has been reported in European and Northeastern American Caucasians but not in African Americans in Baltimore [15,17]. In addition, the association of A*03 to viral clearance has been observed in European Caucasians but not American Caucasians (see Table 4). However, it appears that class I HLA-allele associations are less dependent on ethnicity. For example, four out of six associations in Americans, including the protective association of HLA-A*03, A*11, B57, Cw*01, Cw*05 and the risk association of Cw*04 to HCV infection were statistically significant in both Caucasians and non-Caucasians (see Table 4).

Thus far, three studies, including ours, have shown that DRB1*01, DQB1*05 and the DRB1*01-DQB1*05 haplotype have associations with viral clearance in European and American Caucasians whereas the protective association of HLA-DQB1*03 is exclusive to non-Caucasian Americans in Baltimore and Chicago (see Table 4). In contrast, DQB1*02 and the haplotype DRB1*03-DQB1*02 are susceptible to HCV infection in European and Northeastern American Caucasians in Baltimore [15,17].

Some other associations show additional geographical differences. The risk association of DRB1*07 is significant in Irish Caucasians and perhaps Midwestern Caucasian Americans but not reported in other Europeans and Americans. DRB1*11 was reported to be protective in Italian and Northeastern Americans in Philadelphia rather than in other Europeans and Northeastern Americans in Baltimore and Midwestern Americans (see Table 4). In Northeastern (Baltimore) and Midwestern (Chicago) America, protective association of DQB1*03 was exclusively observed in African and Hispanic Americans but not in Caucasians, while it is significant in British Caucasians and Northwestern American Caucasians but not in African Americans in Philadelphia (see Table 4). The race and geography-dependent differences may explain some inconsistencies in HLA associations with HCV infection.

The recognition and subsequent destruction of infected cells by natural killer (NK) cells and virus-specific cytolytic T lymphocytes (CTL) provide a first line of defense against the virus [21]. In humans with acute spontaneous clearance of HCV, vigorous multi-specific CTL responses are observed early in infection [5,7,22-24]. HLA-class I antigens, together with viral peptides, interact with T cell receptors (TCR) and mediate CD8 cytotoxic activity. HLA-C is believed to be particularly important for the NK cell response because HLA-C molecules are ligands of inhibitory killer cell immunoglobulin-like receptors (KIRs) [25]. The KIR family is composed of activating and inhibitory receptors and effector functions occur only when activating signals overcome inhibitory signals [26]. It has been reported that CD8 cells also express KIRs [26]. The HLA-class I antigens thus can regulate the NK and CD8 cell activity by interacting with different KIRs and TCRs [27,28].

T-helper (Th) CD4 cells, the other key component of adaptive immunity working with HLA-class II antigens together, also play a major role in host defense against viruses and intracellular microbes. Clonal expansion and maintenance of CTL activity depend upon specific Th1 cells [29]. It was reported that the absence of an adequte CD4 response is associated with incomplete control of HCV replication by memory CD8 cells and failure to resolve HCV infection [30]. A protective Th1 response, characterized by Th1 cytokines such as interferon (IFN)-γ, is essential for viral clearance [30]. These qualitatively different immune responses can lead to different outcomes of HCV infection.

There was no significant difference of HLA-allele compositions between different races in our study. The race-based differences should thus be due to different allelic diversity of HLA-gene controlled ligands and their TCRs or KIRs. The genetic difference at either side of the ligand or receptor can lead to an alteration of receptor-ligand binding affinity which subsequently modulates the balance of inhibition and activation of NK cells and T cells [26].

In conclusion, this study revealed, for the first time, that the associations of HLA-A*02 and HLA-DRB1*12 with HCV infection are opposite with different races. In addition, our study indicated that HLA-A*03, Cw*05, DRB1*01/DQB1*05 and DQB1*03 favor viral clearance while HLA-A*01, DRB1*07 and DQB1*02 are risk markers for viral persistence of HCV infection. These results validate previously reported associations, and reveal ethnically and geographically different distribution of HLA-class I and class II genes which affect the outcome of HCV infection. Therefore, recognition of racial differences in HLA associations is likely important in studying the immune response differences and assessing the functionality of hepatitis therapy and vaccines in different races. Thus such race-based studies with larger number of HCV patients are warranted in the future.

## Methods

### Study participants and sample preparation

All samples were obtained with informed consent and approval of the local Institutional Review Board (IRB). The study protocol conforms to the ethical guidelines of 1975 Declaration of Helsinki as reflected in an approval by the IRB. Subjects in this study were participants of a cohort that enrolls injection drug users in the Chicago area with a defined date of HCV acquisition, ethnicity, viral load, and serum levels of alanine aminitransferase. Peripheral blood samples were collected from 105 participants and peripheral blood mononuclear cells (PBMC) were isolated as described previously [20]. Genomic DNA was isolated using a DNA isolation kit (QIAGEN, Valencia, CA, USA). The determination of the presence of HCV-specific antibodies in patients' sera and HCV genotypes was described elsewhere [31]. The detailed information of participants is listed in Table 1.

### HLA typing

Alleles of HLA-A, -C, -DRB1 and -DQB1 loci were determined using the Dynal RELI SSO kits (Dynal Biotech Ltd, UK) according to the manufacturer's instructions. In brief, the test was based on three major processes: PCR target amplification, hybridization of the amplified products to an array of immobilized sequence-specific oligonucleotide (SSO) probes, and detection of the probe-bound amplified product by color formation. The results were analyzed using the software of Pattern Match Program (Dynal Biotech Ltd, UK).

### Statistical analysis

The allele frequencies of the HLA-C, -DRB1 and -DQB1 were calculated by direct counting. Comparisons of allele frequencies were made between those subjects who were chronically infected and those who had sustained viral clearance. Allelic associations with outcomes of HCV infection were analyzed by logistic regression. A multivariable logistic regression model was generated to obtain adjusted odds ratios for independent markers of risk. Magnitude of effect was estimated by odds ratios and their 95% confidence intervals. The statistical significance of the differences in odds ratios in Caucasians and non-Caucasians was assessed by the interaction test.

Pairwise linkage disequilibrium (LD) Δ parameters were calculated using the Burrows formula for composite measure of LD on the Population Genetics Analysis Software PopGene v1.32 .

## Competing interests

The authors declare that they have no competing interests.

## Authors' contributions

JW participated in the study design, supervising experiments, data analysis and manuscript drafting. XK and JS participated in performing the experiments, data collection and analysis. XZ, KB and MO participated in data analysis and MS drafting. BB and RH provided the study subject samples and data collection, finance support. TD participated in statistic analysis and drafting manuscript. SC provided facility and finance support and participated manuscript drafting. LR participated in data analysis and manuscript drafting.
